# Simultaneous determination of 8-oxo-2’-deoxyguanosine and 8-oxo-2’-deoxyadenosine in human retinal DNA by liquid chromatography nanoelectrospray-tandem mass spectrometry

**DOI:** 10.1038/srep22375

**Published:** 2016-03-16

**Authors:** Bin Ma, Meng Jing, Peter W. Villalta, Rebecca J. Kapphahn, Sandra R. Montezuma, Deborah A. Ferrington, Irina Stepanov

**Affiliations:** 1 Masonic Cancer Center, University of Minnesota, Mayo Mail Code 806, 420 Delaware Street SE, Minneapolis, Minnesota 55455, United States; 2 Department of Ophthalmology and Visual Neurosciences, University of Minnesota, Mayo Mail Code 493, 420 Delaware Street SE, Minneapolis, Minnesota 55455, United States; 3 Division of Environmental Health Sciences, University of Minnesota, Mayo Mail Code 807, 420 Delaware Street SE, Minneapolis, Minnesota 55455, United States

## Abstract

Age-related macular degeneration (AMD) is the leading cause of blindness among older adults in the developed world. Oxidative damage to mitochondrial DNA (mtDNA) in the retinal pigment epithelium (RPE) may play a key role in AMD. Measurement of oxidative DNA lesions such as 8-oxo-2’-deoxyguanosine (8-oxo-dG) and 8-oxo-2’-deoxyadenosine (8-oxo-dA) in diseased RPE could provide important insights into the mechanism of AMD development. We have developed a liquid chromatography-nanoelectrospray ionization-tandem mass spectrometry method for simultaneous analysis of 8-oxo-dG and 8-oxo-dA in human retinal DNA. The developed method was applied to the analysis of retinal DNA from 5 donors with AMD and 5 control donors without AMD. In mtDNA, the levels of 8-oxo-dG in controls and AMD donors averaged 170 and 188, and 8-oxo-dA averaged 11 and 17 adducts per 10^6^ bases, respectively. In nuclear DNA, the levels of 8-oxo-dG in controls and AMD donors averaged 0.54 and 0.96, and 8-oxo-dA averaged 0.04 and 0.05 adducts per 10^6^ bases, respectively. This highly sensitive method allows for the measurement of both adducts in very small amounts of DNA and can be used in future studies investigating the pathophysiological role of 8-oxo-dG and 8-oxo-dA in AMD and other oxidative damage-related diseases in humans.

Age-related macular degeneration (AMD) is the leading cause of blindness among the elderly in the developed world. The AMD-associated blindness results from the loss of the retinal pigment epithelium (RPE) and subsequent death of photoreceptors^1^,^2^,^3^. Understanding the underlying mechanisms responsible for the RPE loss and AMD progression is critical for the development of prevention and treatment strategies for this debilitating disease. Recent studies have shown increased mitochondrial DNA (mtDNA) damage in the RPE at stages of AMD preceding macular degeneration and vision loss, and suggested that oxidative damage to mtDNA may play a central role in AMD^2^,^3^,^4^. The particular vulnerability of mtDNA to oxidative stress-induced damage, as compared to nuclear DNA (nDNA), has been previously demonstrated; this can be explained by the production of reactive oxygen species (ROS) in the mitochondria, the lack of protective histones and inefficiency of mtDNA repair mechanisms^4^,^5^,^6^,^7^,^8^.

Oxidative damage to mtDNA and nDNA can occur via incorporation of an oxidized base during DNA polymerization or by oxidation of a normal base that is already part of the DNA. Such modifications lead to the formation of over 20 various modified nucleobase adducts, with the most prominent being 8-oxo-2′-deoxyguanosine (8-oxo-dG, Fig. 1). It is well-established that 8-oxo-dG is a highly mutagenic lesion; it mispairs with A during DNA replication and leads to a GC to AT conversion, which is the most frequent type of spontaneous mutation^9^. In addition to 8-oxo-dG, 8-oxo-2′-deoxyadenosine (8-oxo-dA, Fig. 1) is also detected in the oxidatively damaged DNA and induces mainly A to C transversions^10^. Measurement of these adducts in retinal mtDNA could potentially provide critical insights into the role of oxidative mtDNA damage in AMD.

There have been a few reports of sensitive liquid chromatography-tandem mass spectrometry (LC-MS/MS)-based methodologies for simultaneous analysis of 8-oxo-dG and 8-oxo-dA^11^,^12^,^13^. For instance, Singh *et al*.^13^ reported an accurate and robust column-switching LC-MS/MS method which requires 10–40 μg of hydrolyzed DNA on-column. However, only nanogram amounts of mtDNA can be extracted from human retina^2^, and therefore, a more sensitive and selective method is required for its analysis. Furthermore, in the previous studies the levels of 8-oxo-dA ranged from non-detectable to 10–20 times lower than those of 8-oxo-dG in the same samples^11^,^12^,^14^. On one hand, this suggests that, unlike 8-oxo-dG, 8-oxo-dA might be less prone to artifactual formation and thus serve as a better biomarker for the investigations of the role of oxidative DNA damage in human diseases. On the other hand, the significant difference in the levels of these adducts presents an additional challenge in achieving sufficient sensitivity for their simultaneous analysis in small quantities of human retinal mtDNA.

Our goal in this study was to develop a novel, sensitive, and robust LC-MS/MS method for the simultaneous measurement of 8-oxo-dG and 8-oxo-dA in human RPE mtDNA and to apply this method for the analysis of samples from donors with and without AMD. The ultimate goal of our research is to understand whether the oxidative stress-induced DNA adducts can serve as useful biomarkers for mechanistic studies of AMD development. With that purpose, we also aimed to test our developed method in the analyses of oxidative mtDNA and nDNA damage in neural retina (NR), a neighboring tissue that could potentially serve as a source of larger amounts of DNA in future studies.

## Results

### Development of the analytical procedure

**Figure 1:**
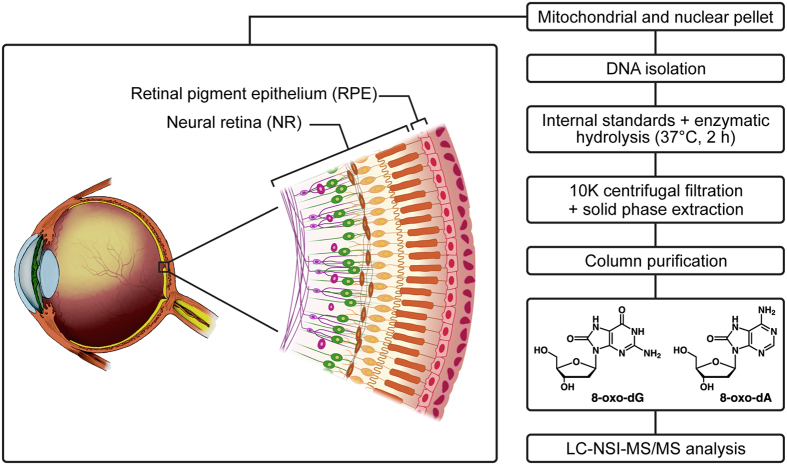
Analytical scheme for the determination of 8-oxo-dG and 8-oxo-dA in human retinal DNA. The image of the eye is modified from the National Eye Institute, National Institutes of Health image library, licensed under CC BY 2.0 (https://creativecommons.org/licenses/by/2.0/).

**Figure 2:**
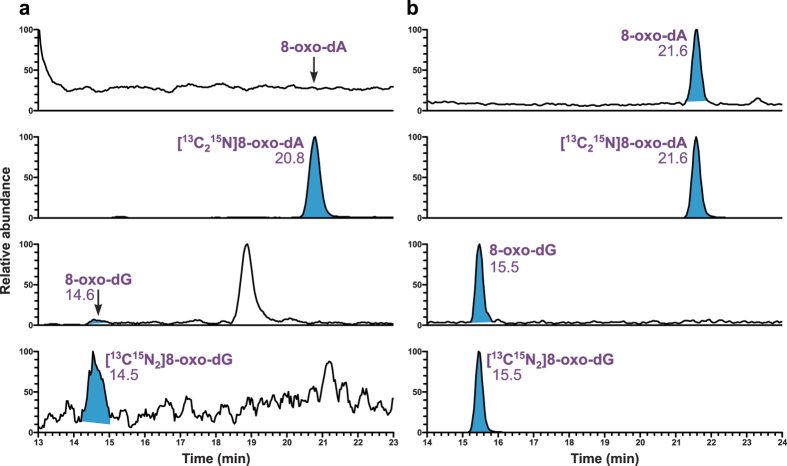
Comparison of chromatograms obtained upon analysis of 8-oxo-dG and 8-oxo-dA by the two LC-MS/MS methods tested in this study. The same mtDNA sample from human retina was analyzed by using (**a**) LC-ESI-MS/MS and (**b**) LC-NSI-MS/MS.

The developed sample preparation procedure for the analysis of 8-oxo-dG and 8-oxo-dA by LC-MS/MS is outlined in Fig. 1. Our initial approach was to use LC-electrospray ionization (ESI)-MS/MS with a capillary flow rate of 10 μL/min for the analysis of the prepared samples. The limit of quantitation (LOQ) of this method was 4 fmol on-column for both adducts in calf thymus DNA (CT-DNA). However, when the method was applied to mtDNA samples from human retina, high background noise levels were observed for both 8-oxo-dG and its internal standard, while 8-oxo-dA was not detected (Fig. 2a). In an effort to optimize the sensitivity of the developed method, the analysis was switched to an LC-nanoelectrospray ionization (NSI)-MS/MS system with a nanoflow rate of 300 nL/min. Analysis of the same mtDNA samples by this method produced clear peaks for both 8-oxo-dG and 8-oxo-dA with significantly improved signal-to-noise ratio (Fig. 2b).

The effect of DNA isolation procedure carried out with or without the addition of various antioxidants on the levels of 8-oxo-dG measured in CT-DNA is summarized in Table 1. Without the addition of antioxidants, the isolation procedure led to DNA oxidation, resulting in a 17% increase in the level of 8-oxo-dG, compared to the levels found in CT-DNA not subjected to this process. The artifactual formation of 8-oxo-dG was not eliminated by adding 2,2,6,6-tetramethyl-piperidinoxyl (TEMPO), while the addition of desferrioxamine (DFO) resulted in lower levels 8-oxo-dG than those found in CT-DNA not subjected to the DNA isolation procedure, suggesting partial loss of the adduct due to decomposition. The use of 8-hydroxyquinoline (8-HQ) during DNA isolation process provided complete protection against oxidation (Table 1), and this antioxidant was further used in DNA isolation from human retinal samples. The absence of artifactual formation of 8-oxo-dG during the hydrolysis and purification of the isolated DNA was evidenced by the absence of [^15^N_5_]8-oxo-dG in samples to which [^15^N_5_]dG was added at the initial step of sample preparation procedure (Supplementary Fig. S1).

### Method Characteristics

The background amount of 8-oxo-dG present in the CT-DNA, as determined during the method development (17.7 fmol/μg DNA, Table 1), was subtracted in the analyses of data for CT-DNA (from the same source) used in method characterization.

**Figure 3:**
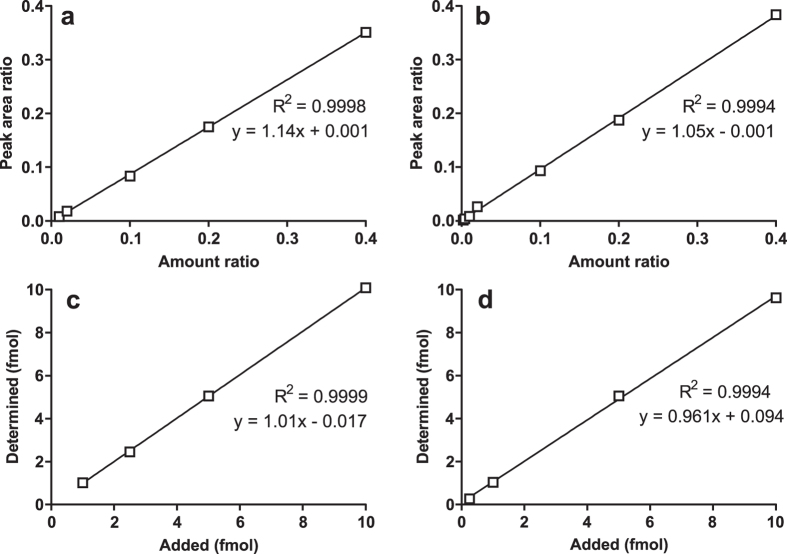
Linearity and accuracy of the assay. (**a**) Linearity of 8-oxo-dG/[^13^C^15^N_2_]8-oxo-dG at constant [^13^C^15^N_2_]8-oxo-dG amount (10 fmol on-column) and 8-oxo-dG ranging from 0.1 to 4 fmol (on-column). (**b**) Linearity of 8-oxo-dA/[^13^C_2_^15^N]8-oxo-dA at constant [^13^C_2_^15^N]8-oxo-dA amount (10 fmol on-column) and 8-oxo-dA ranging from 0.02 to 4 fmol (on-column). (**c**) Correlation between added and measured amounts of 8-oxo-dG. Various amounts of 8-oxo-dG (1, 2.5, 5, 10 fmol) were added to CT-DNA (50 ng), and analyzed by the developed method; 8-oxo-dG present in the CT-DNA was subtracted from each value. (**d**) Correlation between added and measured amounts of 8-oxo-dA. Various amounts of 8-oxo-dA (0.25, 1, 5, 10 fmol) were added to CT-DNA (50 ng), and analyzed by the developed method.

By using the developed LC-NSI-MS/MS method, a limit of detection (LOD) of 0.04 fmol (on-column) was obtained for 8-oxo-dG and 0.01 (on-column) for 8-oxo-dA. The instrument response and the analyte/internal standard ratio were linear in the 0.1−4 fmol (on-column) range of 8-oxo-dG (R^2^ = 0.9998, Fig. 3a) and 0.02−4 fmol (on-column) range of 8-oxo-dA (R^2^ = 0.9994, Fig. 3b). The LOQ of 8-oxo-dG and 8-oxo-dA were 0.1 and 0.02 fmol on-column based on coefficients of variation (%CV) of 4.3% and 5.3%, respectively. The accuracy of measured levels of 8-oxo-dG (expressed as % of added 8-oxo-dG) at 1, 2.5, 5.0 and 10 fmol was 103%, 98.2%, 101% and 101%, respectively; The accuracy of measured levels of 8-oxo-dA (expressed as % of added 8-oxo-dA) at 0.25, 1.0, 5.0 and 10 fmol was 107%, 104%, 101% and 96.3%, respectively. The accuracy exhibited excellent linearity with R^2^ of 0.9999 for 8-oxo-dG (Fig. 3c) and 0.9994 for 8-oxo-dA (Fig. 3d). The inter-day CV of 8-oxo-dG and 8-oxo-dA were 7.7% and 9.6%, respectively. The recoveries of 8-oxo-dG and 8-oxo-dA in the assay were 62.3 ± 5.8% and 71.2 ± 3.2%.

### Application of the developed method: analysis of 8-oxo-dG and 8-oxo-dA in human retinal DNA

**Figure 4:**
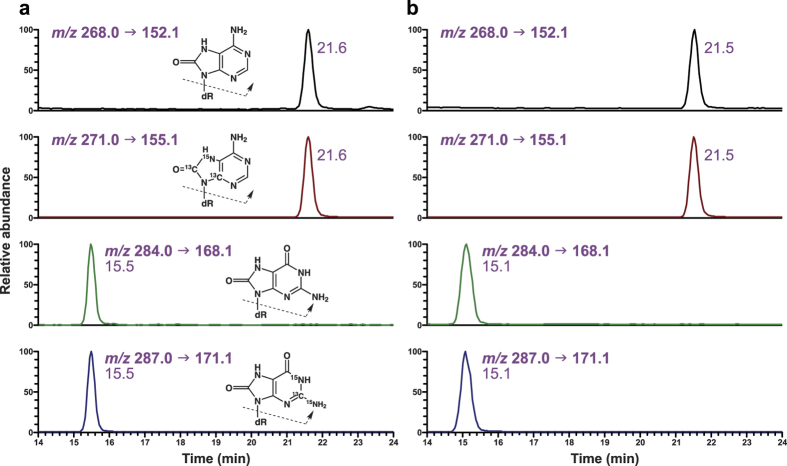
Typical chromatograms obtained upon analysis of 8-oxo-dG and 8-oxo-dA in human RPE mtDNA. Shown are examples of chromatograms from a (**a**) control donor without AMD and (**b**) donor with AMD.

The method was applied to the analysis of human retinal DNA samples from 10 donors. To provide preliminary insights into the potential association between the levels of retinal oxidative mtDNA damage and AMD, samples from 5 control donors without AMD and 5 donors with AMD were selected for this analysis. Typical chromatograms obtained upon analysis of 8-oxo-dG and 8-oxo-dA in mtDNA isolated from RPE of a control donor and a donor with AMD are presented in Fig. 4. The method was further applied to the analysis of mtDNA and nDNA isolated from NR of the same donors. The measured adduct levels in the tested DNA sources from the 10 donors are summarized in Table 2. The yields of DNA in samples from all donors averaged 0.037 ± 0.015 μg in RPE mtDNA, 0.09 ± 0.05 μg in NR mtDNA, and 21 ± 14 μg in NR nDNA (Table 2). In all 10 donors, the levels of 8-oxo-dG in mtDNA averaged 275 ± 122 adducts/10^6^ nucleotides, or 825 ± 366 fmol/μg DNA in RPE, and 83 ± 50 adducts/10^6^ nucleotides, or 249 ± 151 fmol/μg DNA in NR, while the levels were much lower in NR nDNA, averaging 0.75 ± 0.94 adducts/10^6^ nucleotides, or 2.3 ± 2.8 fmol/μg DNA. 8-Oxo-dA was detected in 9 out of 10 RPE mtDNA samples, and the levels averaged 19 ± 12 adducts/10^6^ nucleotides, or 56 ± 36 fmol/μg DNA. The levels of 8-oxo-dA in NR mtDNA averaged 9.2 ± 5.5 adducts/10^6^ nucleotides, or 28 ± 16 fmol/μg DNA, while the levels in NR nDNA samples were much lower, ranging from non-quantifiable to 0.07 adducts/10^6^ nucleotides, or 0.2 fmol/μg DNA.

**Figure 5:**
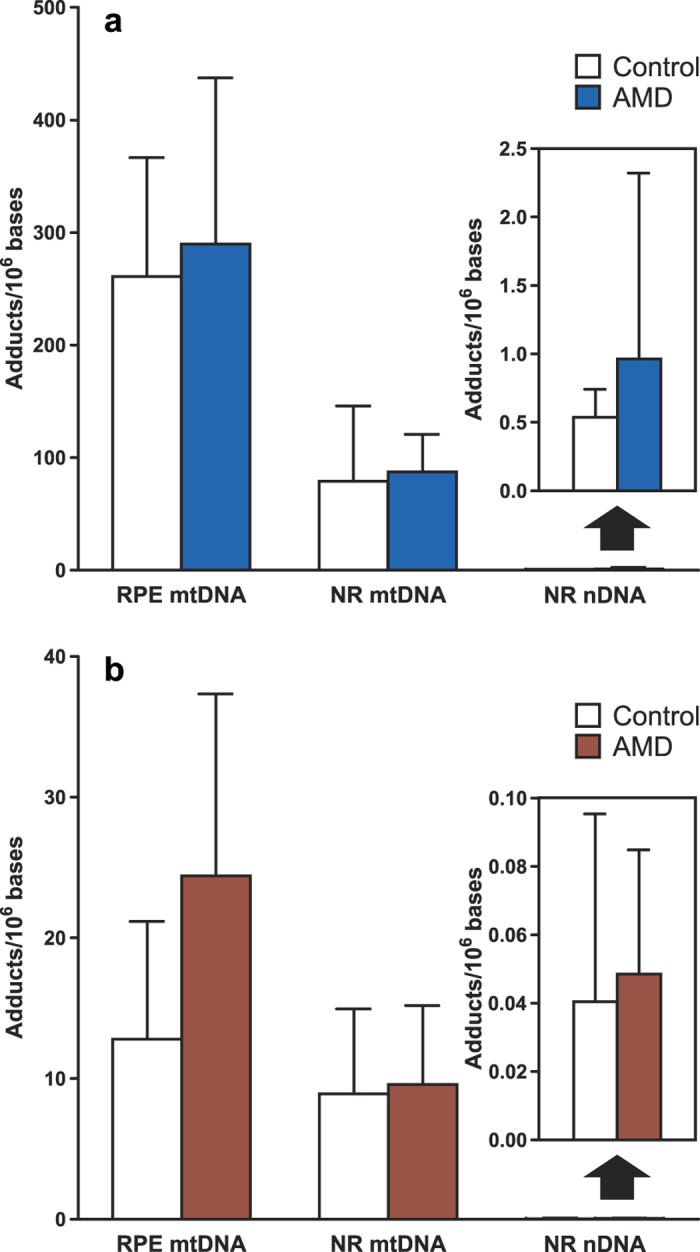
Levels of 8-oxo-dG and 8-oxo-dA in different types of retinal DNA from donors with and without AMD. Data for (**a**) 8-oxo-dG and (**b**) 8-oxo-dA are presented as mean ± SD (5 donors per group). The differences in adduct levels between donors with and without AMD were not statistically significant (P > 0.05).

Figure 5a,b present the average levels of the measured adducts in retinal DNA samples from donors with and without AMD. In RPE mtDNA, the levels of 8-oxo-dG averaged 261 and 290 adducts per 10^6^ nucleotides (Fig. 5a), and 8-oxo-dA averaged 13 and 24 adducts per 10^6^ nucleotides in control and AMD donors (Fig. 5b), respectively. In NR mtDNA, the levels of 8-oxo-dG averaged 79 and 87 adducts per 10^6^ nucleotides, and 8-oxo-dA averaged 9 and 10 adducts per 10^6^ nucleotides in control and AMD donors, respectively. In nDNA, the levels of 8-oxo-dG averaged 0.54 and 0.96 adducts per 10^6^ nucleotides, and 8-oxo-dA averaged 0.04 and 0.05 adducts per 10^6^ nucleotides in control and AMD donors, respectively. While there was an overall tendency towards higher levels of the adducts in samples from AMD donors than in controls, these differences were not statistically significant for any of the comparisons.

## Discussion

Ability to accurately assess the extent of oxidative damage to mtDNA in human retinal tissues is an important tool for the mechanistic studies investigating the role of this damage as a causative factor in the development of age-related macular degeneration and subsequent blindness. In previous studies, the damage to mtDNA from human donor RPE was measured by long-extension polymerase assay^2^,^4^. This assay is based on the interference of certain DNA lesions with the progression of the thermostable DNA polymerase during DNA amplification process^5^, and cannot offer sufficient selectivity for the detection and quantification of specific oxidative modifications, such as 8-oxo-dG and 8-oxo-dA^6^. Other more specific analytical methods that have been previously used for the analysis of 8-oxo-dG and 8-oxo-dA include ^32^P-postlabeling technique^15^, capillary electrophoresis with UV detection^16^, gas chromatography−mass spectrometry^17^, and LC-MS/MS^11^,^12^,^14^, none of which could offer sufficient selectivity and/or sensitivity for the analysis of these adducts in the minimal amounts of mtDNA available from human retinal samples. In this study, we developed a novel LC-NSI-MS/MS method for simultaneous determination of 8-oxo-dG and 8-oxo-dA and successfully applied this sensitive and specific method to the analysis of retinal DNA from human donors with and without AMD.

Sensitivity and selectivity of an LC-MS/MS-based analytical assay depends on a variety of factors, ranging from the efficiency of sample purification to remove the components capable of causing ion suppression, to the particulars of chromatographic conditions, to the resolution capacity and selectivity of the mass-spectrometer coupled to the LC system. During the method development, we initially developed an efficient solid-phase extraction procedure (Strata-X), which allowed us to conduct the analysis of the prepared samples by LC-ESI-MS/MS at a capillary flow rate of 10 μL/min, achieving on-column LOQ of 4 fmol for both adducts in CT-DNA. However, when this method was applied to the analysis of human retinal mtDNA, high background noise was observed in 8-oxo-dG chromatograms, while 8-oxo-dA was not detected. To increase the sensitivity of the method, we switched to nano-ESI with a flow rate of 300 nL/min, which has been applied in our previous study on another DNA adduct, 3-(2-deoxy-*β*-derythropentafuranosyl)pyrimido[1,2-*α*]purin-10(3*H*)-one deoxyguanosine (M_1_dG), a DNA adduct formed by malondialdehyde, and demonstrated higher sensitivity compared to capillary-ESI^18^. While switching to nano-ESI led to significant reduction of the background noise and allowed us to detect both adducts, substantial ion suppression was observed for both analytes. This was also observed in our previous study on M_1_dG^18^, and is not surprising given that nano-ESI has been reported to suffer from ion suppression due to insufficient sample purification^19^. In this study, we subjected samples to column purification (Fig. 1), which proved to be a critical step leading to substantial removal of ion suppression and subsequent significant improvement of MS signal. By using this optimized method, we reached an on-column LOQ of 0.1 fmol for 8-oxo-dG and 0.02 fmol for 8-oxo-dA, which constitutes a 40-fold and 50-fold improvement, respectively, compared with the original capillary-ESI method.

Artifactual formation of 8-oxo-dG via the oxidation of dG during sample processing and analysis is a major concern^20^,^21^. For instance, there are substantial discrepancies in the endogenous 8-oxo-dG levels measured in different studies, with the reported levels in mtDNA varying by more than 1,000-fold^20^. While the effect of different methodologies that often lack specificity could have played a role in these discrepancies, the artifactual formation of the adduct during sample preparation and analysis is most likely a major contributor. In our study, we took precautions to prevent the artifactual DNA oxidation throughout the whole analytical procedure, including the DNA isolation, enzymatic hydrolysis, sample purification, and analysis. Artifactual formation of 8-oxo-dA did not present a concern, because this adduct was not detected in CT-DNA during method development; therefore, we limited our efforts to monitoring and prevention of the potential dG oxidation. The use of 8-HQ during DNA isolation led to complete prevention of artifactual 8-oxo-dG formation, in agreement with the reported use of this antioxidant by Singh *et al*.^13^ We also established that subsequent steps in sample preparation procedure do not lead to artifactual 8-oxo-dG formation, as evidenced by the absence of [^15^N_5_]8-oxo-dG in samples that were mixed with [^15^N_5_]dG prior to being subjected to enzymatic hydrolysis followed by the developed purification protocol. Another potential source of artifactual production may be generated by the oxidation of excessive dG in the sample during mass spectrometry analysis. It has been reported that dG was partly oxidized in the ion source of the mass spectrometer after elution from HPLC column^22^,^23^. However, dG and 8-oxo-dG have different retention time on HPLC column (see Supplementary Fig. S2) and therefore, the chromatographic peak of 8-oxo-dG formed in the ion source would not interfere with that of 8-oxo-dG present in sample. Nevertheless, complete removal of dG from the sample is preferable in order to prevent any potential interference due to its oxidation in the ion source. In our method, efficient separation of dG and 8-oxo-dG during the column purification step (see Supplementary Fig. S2) allowed removing dG from the samples and avoid concerns about such potential interference.

In this study, the analysis of 8-oxo-dG and 8-oxo-dA in various types of retinal DNA from the same donors was carried out primarily to test the applicability of the developed methodology to the analyses of small amounts of retinal DNA in future investigations of the role of oxidative stress in AMD. Despite this limited purpose and the small number of samples analyzed, the observed differences in the adduct levels among the different types of retinal DNA offer an insight into the potential utility of these adducts in providing mechanistic support for the hypothesis that oxidative mtDNA damage in RPE is a key step in the progression of AMD. In agreement with this hypothesis, the relative levels of 8-oxo-dG and 8-oxo-dA in RPE mtDNA were higher (8-oxo-dG, P = 0.0002; 8-oxo-dA, P = 0.037) than those in NR mtDNA (Fig. 5). The almost 2-fold difference in the levels of 8-oxo-dA measured in RPE mtDNA from donors with and without AMD, while not statistically significant (P = 0.13), is also of note. It is possible that, due to the lack of substantial background levels of 8-oxo-dA in DNA, this adduct might serve as a better biomarker than 8-oxo-dG in characterizing the AMD-associated oxidative DNA damage. Furthermore, much higher levels of the measured adducts were found in mtDNA than in nDNA isolated from the same retina samples (Fig. 5). This observation agrees with previous findings that mtDNA is more susceptible to DNA damage than nDNA, potentially due to exposure to high levels of ROS produced during oxidative phosphorylation, the lack of protective histones, and inefficient DNA repair mechanisms^24^,^25^,^26^. Higher levels of specific DNA adducts in mtDNA as compared to nDNA have been also reported. For instance, Mecocci *et al*. reported that the levels of 8-oxo-dG in mtDNA from human aging brain were 10-fold higher than those in nDNA^27^. In another study, the levels of 8-oxo-dG in mtDNA in both the heart and the brain tissues from six mammalian species were 3- to 9-fold higher than the levels in nDNA from the corresponding tissues^28^. Lastly, our previous analysis of tissues from F344 rats treated with tobacco-specific nitrosamines also demonstrated higher levels of the nitrosamine-derived DNA adducts in mtDNA than nDNA^24^. Together, these findings suggest that the damage to mitochondrial DNA due to oxidative stress or environmental exposures could play an important role in human chronic diseases and that measurement of mtDNA adducts is a potential critical tool for the relevant mechanistic and molecular epidemiology studies. In the case of AMD, the next step is to apply our developed methodology to measure 8-oxo-dG and 8-oxodA in a larger sample of human retinal samples from donors with different AMD stages, to potentially provide important mechanistic insights into the role of oxidative damage to mtDNA in the development of this disease.

In summary, we developed a novel and highly sensitive LC-NSI-MS/MS method for simultaneous quantitation of 8-oxo-dG and 8-oxo-dA in extremely small amounts of human DNA, and successfully applied this method to the analysis of retinal mtDNA and nDNA from 10 donors. This methodology can be applied in future studies aimed at understanding the pathophysiological role of 8-oxo-dG and 8-oxo-dA in AMD and other oxidative damage-related diseases.

## Materials and Methods

### Chemicals and enzymes

8-Oxo-dG, [^13^C^15^N_2_]8-oxo-dG, 8-oxo-dA, and [^13^C_2_^15^N]8-oxo-dA were purchased from Toronto Research Chemicals (North York, Ontario, Canada). Reagents and enzymes for DNA isolation were obtained from Qiagen Sciences (Germantown, MD). CT-DNA was purchased from Worthington Biochemical Corporation (Lakewood, NJ). All other chemicals and solvents were purchased from Sigma-Aldrich Chemical Co. (Milwaukee, WI).

### Donors and retina sample collection

De-identified donor eyes were obtained from the Minnesota Lions Eye Bank, which receives these samples from deceased individuals with the consent of the donor and donor’s family for use in medical research in accordance with the Declaration of Helsinki. The Minnesota Lions Eye Bank is licensed by the Eye Bank Association of America (accreditation #0015204) and accredited by the FDA (FDA Established Identifier 3000718538). Donor tissue is exempt from the process of Institutional Review Board approval. Processing of donor tissue and evaluation of the donor’s stage of AMD was as previously outlined^4^. Analysis was performed on donors without AMD (control) and donors at the intermediate stage of AMD (diseased). Control donors had no clinically observable eye disease. To eliminate from the potential effect of other eye diseases on oxidative damage to DNA, exclusion criteria for donors used in this study included a history of diabetes or glaucoma, clinical symptoms of diabetic retinopathy, advanced glaucoma, and myopic degeneration.

### Nuclear and mitochondrial enrichment

RPE and NR harvested from donor eyes were re-suspended with 150 μL mitochondrial enrichment buffer containing 0.5% Nonidet P-40, 20 mM 4-(2-hydroxyethyl)-1-piperazineethanesulfonic acid (pH 7.5), 10 mM KCl, 1.5 mM MgCl_2_, 250 mM sucrose, 1 mM EDTA-ethylenediamine tetraacetic acid, and 1 mM EGTA-ethylene glycol tetraacetic acid. Homogenization of RPE samples involved two freeze/thaw cycles with liquid nitrogen followed by passage through a 28-gauge needle six times. Homogenization of NR samples was carried out in a glass dounce homogenizer. Cellular debris and nuclei in the homogenized samples were precipitated by centrifugation at 600 *g* for 15 min at 4 °C. The precipitate was discarded in the case of RPE because of our initial focus exclusively on mtDNA from this tissue. As the study evolved, however, we decided to save the precipitate during NR processing, and used it to isolate nDNA from these samples. For both RPE and NR, after the precipitation of nuclear fraction supernatant was removed and placed in a new tube. Mitochondria were pelleted by centrifugation at 13,000 *g* for 15 min at 4 °C.

### DNA isolation from human retina

DNA isolation from mitochondrial and nuclear pellets was performed using the protocol available in Qiagen QIAamp DNA Mini Kit (Qiagen, Valencia, CA) with several modifications. To prevent the artifactual formation of 8-oxo-dG during the DNA isolation, 8-HQ as an antioxidant^13^ was added to all the extraction buffers to a final concentration of 0.35 mM. Briefly, 200 μL of ATL buffer was added to the pellet and proteinase K (10 μL of 20 mg/mL solution) was added and incubated at 56 °C for 1 h with gentle shaking. After the incubation, 4 μL of RNase A solution (100 mg/mL) was added, and the sample was incubated at room temperature for 2 min, followed by the addition of 200 μL of AL buffer and incubating at 70 °C for 10 min. The sample was then mixed with 200 μL EtOH, and the mixture was loaded onto a Mini spin column and centrifuged at 6,000 *g* for 1 min. The column was washed with 500 μL of AW1 buffer and 500 μL of AW2 buffer sequentially, and the DNA was finally eluted with 400 μL of nuclease free H_2_O.

### DNA hydrolysis and adduct enrichment

The DNA solution was mixed with 40 μL 400 mM Tris buffer (pH8.5) containing 100 mM MgCl_2_, followed by addition of 500 fmol [^13^C^15^N_2_]8-oxo-dG, 500 fmol [^13^C_2_^15^N]8-oxo-dA and 1,000 fmol [^15^N_5_]dG as internal standards. The resulting solution was then mixed with deoxyribonuclease I (1 unit), phosphodiesterase I (0.005 units) and alkaline phosphatase (30 units), and then incubated at 37 °C for 2 h. After incubation, 25 μL of hydrolysate was taken for the analysis of dG by LC-MS/MS (see Supplementary Fig. S3), which was used to calculate the amount of DNA as described previously^29^,^30^. The remaining volume of hydrolysate was filtered by centrifugal filtration (Ultracel 10 K, Millipore) before loading on Strata-X cartridge (30 mg, Phenomenex) activated with 1 mL MeOH and 1 mL H_2_O. The cartridge was washed with 0.8 mL of H_2_O and 0.5 mL of 3% MeOH sequentially, and finally eluted with 1.5 mL of 35% MeOH. The 35% MeOH fraction containing analytes was collected and concentrated to dryness in a centrifugal evaporator. The residue was re-dissolved in 10 μL deionized H_2_O and subjected to column purification on an Agilent 1100 HPLC system equipped with a Synergi Hydro-RP column (4 μm, 250 × 0.5 mm, Phenomenex). The mobile phase consisted of 10 mM ammonium formate (pH 4.2) and MeOH, with a gradient from 10 to 30% MeOH within 20 min, increased to 60% MeOH over 5 min, then returned to 10% MeOH in 1 min and held for 15 min at this composition, at a flow rate of 10 μL/min. The detection wavelength was set at 254 nm and the column temperature was maintained at 30°C. Thymidine (4 μg/mL) and M_1_dG (2 μg/mL), which have similar retention times to that of 8-oxo-dG (~17.1 min) and 8-oxo-dA (~24.4 min), respectively, were used as UV markers. The fractions eluting at 16–18 min and 24–26 min were collected (see Supplementary Fig. S2), evaporated to dryness, and re-dissolved in H_2_O prior to mass spectrometry analysis.

CT-DNA was used to assess possible artifactual formation during the DNA isolation. Briefly, 1 μg CT-DNA was dissolved in 200 μL H_2_O and processed by following the DNA isolation protocol mentioned above in the absence or presence of different antioxidants, including TEMPO (20 μM)^31^, DFO (5 mM)^21^, and 8-HQ (0.35 mM)^13^, in all the buffers used for DNA isolation. The isolated DNA samples were hydrolyzed and purified as described above. The levels of 8-oxo-dG and 8-oxo-dA were measured and compared with the levels in CT-DNA directly hydrolyzed without the DNA isolation step. Since no 8-oxo-dA was detected in CT-DNA samples, the investigation of artifactual formation was focused on 8-oxo-dG.

To assess possible artifactual formation of 8-oxo-dG during the sample preparation, 500 fmol of [^15^N_5_]dG was added to CT-DNA before enzymatic hydrolysis, and the sample was enzymatically hydrolyzed and purified as described above. The possible artifactual formation of [^15^N_5_]8-oxo-dG was monitored.

### LC-ESI-MS/MS

The LC-ESI-MS/MS analysis was carried out on a TSQ Vantage triple quadrupole mass spectrometer (Thermo Scientific, Waltham, MA) interfaced with an Agilent 1100 capillary HPLC system (Agilent, Palo Alto, CA). Analysis was performed on a Synergi Hydro-RP column (4 μm, 250 × 0.5 mm, Phenomenex) at a flow rate of 10 μL/min with the temperature maintained at 30 °C. Sample injection volume was 5 μL. The mobile phase consisted of 10 mM ammonium formate (pH 4.2) and MeOH with a linear gradient from 8 to 22% MeOH over a period of 18 min, increased to 95% MeOH over 5 min, and then returned to 8% MeOH followed by 15 min re-equilibration. The ESI source was operated in positive ion mode, monitoring *m/z* 284.2 [M + H]^+^→168.2 [C_5_H_6_N_5_O_2_]^+^ for 8-oxo-dG, *m/z* 268.2 [M + H]^+^→152.2 [C_5_H_6_N_5_O]^+^ for 8-oxo-dA, and their corresponding ions at *m/z* 287.2→171.2 for [^13^C^15^N_2_]8-oxo-dG and *m/z* 271.2→155.2 for [^13^C_2_^15^N]8-oxo-dA. The collision gas was Ar at 1 mTorr with collision energy of 16 eV. The quadrupoles were operated at a resolution of 0.2 (Q1) and 0.7 (Q3) Da.

The dG analysis was carried out on the same LC-ESI-MS/MS system. Analysis was performed on a Synergi Hydro-RP column (4 μm, 250 × 0.5 mm, Phenomenex) at a flow rate of 10 μL/min with the temperature maintained at 30 °C. Sample injection volume was 8 μL. The mobile phase consisted of 10 mM ammonium formate (pH 4.2) and MeOH with a linear gradient from 8 to 22% MeOH over a period of 18 min, increased to 95% MeOH over 5 min, and then returned to 8% MeOH followed by 15 min re-equilibration. The ESI source was operated in positive ion mode, monitoring *m/z* 268.2 [M + H]^+^→152.2 [C_5_H_6_N_5_O]^+^ for dG, and its corresponding ion at *m/z* 273.1→157.1 for [^15^N_5_]dG, respectively. The collision gas was Ar at 1 mTorr with collision energy of 16 eV. The quadrupoles were operated at a resolution of 0.7 Da for both Q1 and Q3.

### LC-NSI-MS/MS

The LC-NSI-MS/MS was performed on a TSQ Quantiva triple quadrupole mass spectrometer (Thermo Scientific, Waltham, MA) interfaced with a nanoACQUITY UPLC (Waters, Milford, MA) system using nanoelectrospray ionization. The analysis was performed using a capillary column (75 μm ID, 19 cm length, 15 μm orifice) created by hand packing a commercially available fused-silica emitter (New Objective, Woburn MA) with Synergi Hydro-RP bonded separation media (Phenomenex, Torrance, CA). The mobile phase consisted of 5 mM ammonium formate (pH 4.2) and MeOH. A 5 μL injection loop was used and the sample (4 μL) was loaded onto the capillary column with a 300 nL/min flow at the initial conditions for 10 min. Separation on the capillary column was performed using a linear gradient at a flow rate of 300 nL/min with increasing MeOH from 0 to 35% over 10 min, followed by ramping to 90% MeOH within 1 min and holding at this composition for an additional 4 min. The gradient was then returned to 0% MeOH (initial conditions) in 1 min and the system was re-equilibrated at this mobile phase composition for 15 min before the next injection. The nanoelectrospray source voltage was set at 2 kV. The capillary temperature was 400 °C, and the RF Lens was set at 70 V. The mass transitions for monitoring the analytes were the same as in LC-ESI-MS/MS. The collision gas was Ar at 1 mTorr with collision energy of 12 eV, and the quadrupoles were operated at a resolution of 0.7 Da for both Q1 and Q3.

The quantitation of 8-oxo-dG and 8-oxo-dA was based on the peak area ratio of the analytes to their corresponding isotope-labeled internal standards, the constructed calibration curves, and the amount of internal standards added. Calibration curves were constructed for each analyte before each analysis using a series of standard solutions of analytes and internal standards. The calibration standard solutions contained a constant amount of internal standards (10 fmol on-column) and varying amounts of analytes (0.1, 0.2, 1, 2, and 4 fmol on-column for 8-oxo-dG, and 0.02, 0.1, 0.2, 1, 2, and 4 fmol on-column for 8-oxo-dA).

### Method characterization and sample analysis

Accuracy was determined by adding different amounts of analytes (1, 2.5, 5, and 10 fmol for 8-oxo-dG, and 0.25, 1, 5, and 10 fmol for 8-oxo-dA) and internal standards (50 fmol each) to 50 ng of CT-DNA in 0.4 mL 40 mM Tris buffer (pH8.5) containing 10 mM MgCl_2_, followed by hydrolysis and purification as described above. Samples at each level of added analytes were analyzed in triplicate^32^. To characterize method precision, 5 fmol of the analytes and 50 fmol of internal standards were added to 50 ng of CT-DNA, followed by the previously described protocol. The precision was determined as intra-day and inter-day CV, which were calculated based on the analyses of three aliquots of the samples on three separate days. 8-oxo-dG was present in CT-DNA, and its level was quantified and subtracted from the levels of 8-oxo-dG measured in the samples during the method characterization. No 8-oxo-dA was detected in the CT-DNA samples used in this study.

The LOD was determined using standard solutions of 8-oxo-dG and 8-oxo-dA. The LOQ was established in CT-DNA samples by adding both analytes (0.01, 0.02, 0.05 and 0.1 fmol) and internal standards (50 fmol) to CT-DNA samples, followed by hydrolysis and purification, and analyzing each sample in triplicate. The LOQ was defined by identification of the lowest analyte level that produced a CV lower than 5%^33^.

Recovery was determined by comparing the results of samples in which [^13^C^15^N_2_]8-oxo-dG and [^13^C_2_^15^N]8-oxo-dA (50 fmol each) were added to 50 ng CT-DNA at the beginning or at the end of the sample preparation procedure (n = 5)^18^. All data are presented as mean ± standard deviation (SD). Two-tailed unpaired *t*-test was used for two group comparison. A *p* value less than 0.05 was considered significant.

## Additional Information

**How to cite this article**: Ma, B. *et al*. Simultaneous determination of 8-oxo-2'-deoxyguanosine and 8-oxo-2'-deoxyadenosine in human retinal DNA by liquid chromatography nanoelectrospray-tandem mass spectrometry. *Sci. Rep.* **6**, 22375; doi: 10.1038/srep22375 (2016).

## Supplementary Material

Supplementary Information

## Figures and Tables

**Table 1:** Levels of 8-oxo-dG in CT-DNA treated in the presence of different antioxidants.

**CT-DNA**	**Antioxidant**	**8-oxo-dG (fmol/μg DNA)**
Without DNA isolation		17.7 ± 1.65
With DNA isolation	No antioxidant	20.7 ± 0.54
	TEMPO	21.4 ± 4.44
	DFO	13.8 ± 4.89
	8-HQ	18.2 ± 0.38

**Table 2:** Levels of 8-oxo-dG and 8-oxo-dA in human retinal DNA.

**Donor #**	**RPE Mitochondria**			**NR Mitochondria**			**NR Nuclei**		
	**DNA (μg)**	**Adducts/10**^**6**^ **nucleotides**		**DNA (μg)**	**Adducts/10**^**6**^ **nucleotides**		**DNA (μg)**	**Adducts/10**^**6**^ **nucleotides**	
		**8-oxo-dG**	**8-oxo-dA**		**8-oxo-dG**	**8-oxo-dA**		**8-oxo-dG**	**8-oxo-dA**
Controls									
1	0.031	329	22.3	0.010	194	7.94	22.4	0.66	0.07
2	0.037	226	9.41	0.115	19.9	0.98	20.3	0.67	0.05
3	0.011	405	<LOQ	0.020	69.4	17.9	4.82	0.63	<LOQ
4	0.055	137	15.6	0.182	52.3	9.02	2.96	0.54	<LOQ
5	0.051	207	16.4	0.092	58.3	8.83	31.0	0.18	<LOQ
Donors with AMD									
1	0.053	208	18.1	0.094	88.6	6.44	26.8	0.49	0.06
2	0.033	231	22.0	0.108	142	19.4	23.1	0.43	0.04
3	0.026	241	24.0	0.135	80.9	8.90	28.5	0.45	0.03
4	0.047	215	11.9	0.063	71.5	7.51	1.44	3.37	<LOQ
5	0.022	553	46.0	0.094	52.6	5.59	44.1	0.08	0.02
Average^a^	0.037	275	18.6	0.091	83.0	9.24	20.5	0.75	0.045
SD	0.015	122	11.9	0.051	50.3	5.49	13.7	0.94	0.045

^a^Average values are for controls and donors with AMD combined, because there was no statistically significant difference in DNA adduct levels between the two groups. To calculate average values, 1/2 of LOQ (0.8 fmol/sample) was used for samples in which 8-oxo-dA was not quantifiable. For each sample, LOQ was divided by DNA yield, and half of that value was used.

## References

[b1] Klein R. . Fifteen-year cumulative incidence of age-related macular degeneration: the Beaver Dam Eye Study. *Ophthalmology*. 114, 253–262 (2007). 17270675 10.1016/j.ophtha.2006.10.040

[b2] Karunadharma P. P., Nordgaard C. L., Olsen T. W. & Ferrington D. A. Mitochondrial DNA damage as a potential mechanism for age-related macular degeneration. *Invest. Ophthalmol. Vis. Sci*. 51, 5470–5479 (2010). 20505194 10.1167/iovs.10-5429 PMC3061495

[b3] Lin H. . Mitochondrial DNA damage and repair in RPE associated with aging and age-related macular degeneration. *Invest. Ophthalmol. Vis. Sci*. 52, 3521–3529 (2011). 21273542 10.1167/iovs.10-6163 PMC3109040

[b4] Terluk M. R. . Investigating mitochondria as a target for treating age-related macular degeneration. *J. Neurosci*. 35, 7304–7311 (2015). 25948278 10.1523/JNEUROSCI.0190-15.2015 PMC4420790

[b5] Santos J. H., Mandavilli B. S. & Van Houten B. Measuring oxidative mtDNA damage and repair using quantitative PCR. *Methods Mol. Biol*. 197, 159–176 (2002). 12013794 10.1385/1-59259-284-8:159

[b6] Kovalenko O. A. & Santos J. H. In *Current Protocols in Human Genetics*. Suppl. 62, 19.1.1-19.1.13 (2009). 10.1002/0471142905.hg1901s62 19582765

[b7] Yakes F. M. & Van Houten B. Mitochondrial DNA damage is more extensive and persists longer than nuclear DNA damage in human cells following oxidative stress. Proc. Natl. Acad. Sci. USA 94, 514–519 (1997). 9012815 10.1073/pnas.94.2.514 PMC19544

[b8] Clayton D. A. Transcription of the mammalian mitochondrial genome. *Annu. Rev. Biochem*. 53, 573–594 (1984). 6383200 10.1146/annurev.bi.53.070184.003041

[b9] Dizdaroglu M., Jaruga P., Birincioglu M. & Rodriguez H. Free radical-induced damage to DNA: mechanisms and measurement. *Free Radic. Biol. Med*. 32, 1102–1115 (2002). 12031895 10.1016/s0891-5849(02)00826-2

[b10] Kalam M. A. . Genetic effects of oxidative DNA damages: comparative mutagenesis of the imidazole ring-opened formamidopyrimidines (Fapy lesions) and 8-oxo-purines in simian kidney cells. *Nucleic Acids Res*. 34, 2305–2315 (2006). 16679449 10.1093/nar/gkl099 PMC1458282

[b11] Podmore I. D., Cooper D., Evans M. D., Wood M. & Lunec J. Simultaneous measurement of 8-oxo-2′-deoxyguanosine and 8-oxo-2′-deoxyadenosine by HPLC-MS/MS. *Biochem. Biophys. Res. Commun*. 277, 764–770 (2000). 11062026 10.1006/bbrc.2000.3752

[b12] Weimann A., Belling D. & Poulsen H. E. Measurement of 8-oxo-2′-deoxyguanosine and 8-oxo-2′-deoxyadenosine in DNA and human urine by high performance liquid chromatography-electrospray tandem mass spectrometry. *Free Radic. Biol. Med*. 30, 757–764 (2001). 11275475 10.1016/s0891-5849(01)00462-2

[b13] Singh R. . Effects of environmental air pollution on endogenous oxidative DNA damage in humans. *Mutat. Res*. 620, 71–82 (2007). 17434188 10.1016/j.mrfmmm.2007.02.024

[b14] Gokce G. . Glutathione depletion by buthionine sulfoximine induces oxidative damage to DNA in organs of rabbits *in vivo*. *Biochemistry*. 48, 4980–4987 (2009). 19374446 10.1021/bi900030z

[b15] Gupta R. C. & Arif J. M. An improved (32)P-postlabeling assay for the sensitive detection of 8-oxodeoxyguanosine in tissue DNA. *Chem. Res. Toxicol*. 14, 951–957 (2001). 11511168 10.1021/tx000131d

[b16] Kvasnicova V., Samcova E., Jursova A. & Jelinek I. Determination of 8-hydroxy-2′-deoxyguanosine in untreated urine by capillary electrophoresis with UV detection. *J. Chromatogr. A*. 985, 513–517 (2003). 12580520 10.1016/s0021-9673(02)01527-3

[b17] Lin H. S. . A high-throughput and sensitive methodology for the quantification of urinary 8-hydroxy-2′-deoxyguanosine: measurement with gas chromatography-mass spectrometry after single solid-phase extraction. *Biochem. J*. 380, 541–548 (2004). 14992687 10.1042/BJ20040004 PMC1224185

[b18] Ma B., Villalta P. W., Balbo S. & Stepanov I. Analysis of a malondialdehyde-deoxyguanosine adduct in human leukocyte DNA by liquid chromatography nanoelectrospray-high-resolution tandem mass spectrometry. *Chem. Res. Toxicol*. 27, 1829–1836 (2014). 25181548 10.1021/tx5002699 PMC4203394

[b19] Schmidt A., Karas M. & Dulcks T. Effect of different solution flow rates on analyte ion signals in nano-ESI MS, or: when does ESI turn into nano-ESI? *J. Am. Soc. Mass Spectrom*. 14, 492–500 (2003). 12745218 10.1016/S1044-0305(03)00128-4

[b20] Marcelino L. A. & Thilly W. G. Mitochondrial mutagenesis in human cells and tissues. *Mutat. Res*. 434, 177–203 (1999). 10486591 10.1016/s0921-8777(99)00028-2

[b21] Chao M. R., Yen C. C. & Hu C. W. Prevention of artifactual oxidation in determination of cellular 8-oxo-7,8-dihydro-2′-deoxyguanosine by isotope-dilution LC-MS/MS with automated solid-phase extraction. *Free Radic. Biol. Med*. 44, 464–473 (2008). 17983606 10.1016/j.freeradbiomed.2007.10.003

[b22] Singh R., McEwan M., Lamb J. H., Santella R. M. & Farmer P. B. An improved liquid chromatography/tandem mass spectrometry method for the determination of 8-oxo-7,8-dihydro-2′-deoxyguanosine in DNA samples using immunoaffinity column purification. *Rapid Commun. Mass Spectrom*. 17, 126–134 (2003). 12512091 10.1002/rcm.883

[b23] Ravanat J. L., Duretz B., Guiller A., Douki T. & Cadet J. Isotope dilution high-performance liquid chromatography-electrospray tandem mass spectrometry assay for the measurement of 8-oxo-7,8-dihydro-2′-deoxyguanosine in biological samples. J. Chromatogr. B. Biomed. Sci. Appl . 715, 349–356 (1998). 9792521 10.1016/s0378-4347(98)00259-x

[b24] Stepanov I. & Hecht S. S. Mitochondrial DNA adducts in the lung and liver of F344 rats chronically treated with 4-(methylnitrosamino)-1-(3-pyridyl)-1-butanone and (S)-4-(methylnitrosamino)-1-(3-pyridyl)-1-butanol. *Chem. Res. Toxicol*. 22, 406–414 (2009). 19166332 10.1021/tx800398x PMC2664261

[b25] Santos R. X. . Mitochondrial DNA oxidative damage and repair in aging and Alzheimer’s disease. *Antioxid. Redox Signal*. 18, 2444–2457 (2013). 23216311 10.1089/ars.2012.5039 PMC3671662

[b26] Mambo E. . Electrophile and oxidant damage of mitochondrial DNA leading to rapid evolution of homoplasmic mutations. Proc. Natl. Acad. Sci. USA 100, 1838–1843 (2003). 12578990 10.1073/pnas.0437910100 PMC149920

[b27] Mecocci P. . Mitochondrial membrane fluidity and oxidative damage to mitochondrial DNA in aged and AD human brain. *Mol. Chem. Neuropathol*. 31, 53–64 (1997). 9271005 10.1007/BF02815160

[b28] Barja G. & Herrero A. Oxidative damage to mitochondrial DNA is inversely related to maximum life span in the heart and brain of mammals. *FASEB J*. 14, 312–318 (2000). 10657987 10.1096/fasebj.14.2.312

[b29] Lao Y., Yu N., Kassie F., Villalta P. W. & Hecht S. S. Formation and accumulation of pyridyloxobutyl DNA adducts in F344 rats chronically treated with 4-(methylnitrosamino)-1-(3-pyridyl)-1-butanone and enantiomers of its metabolite, 4-(methylnitrosamino)-1-(3-pyridyl)-1-butanol. *Chem. Res. Toxicol*. 20, 235–245 (2007). 17305407 10.1021/tx060207r PMC2518979

[b30] Gadaleta G. . The complete nucleotide sequence of the Rattus norvegicus mitochondrial genome: cryptic signals revealed by comparative analysis between vertebrates. *J. Mol. Evol*. 28, 497–516 (1989). 2504926 10.1007/BF02602930

[b31] Boysen G. . Analysis of 8-oxo-7,8-dihydro-2′-deoxyguanosine by ultra high pressure liquid chromatography-heat assisted electrospray ionization-tandem mass spectrometry. J. Chromatogr. B. Biomed. Sci. Appl . 878, 375–380 (2010). 10.1016/j.jchromb.2009.12.004 PMC5085061 20022307

[b32] U.S. Food and Drug Administration, *Guidance for Industry: Bioanalytical Method Validation* (2001). Available at: www.fda.gov/downloads/drugs/guidancecomplianceregulatoryinformation/guidances/ucm070107.pdf. (Accessed: 4th November 2015).

[b33] Dolan J. W. In Calibration Curves, Part II: What are the Limits? LCGC North America, Vol. 27, 306–307 (Dolan, 2009).

