# Supplementary material for: Simultaneous determination of 8-oxo-2’-deoxyguanosine and 8-oxo-2’-deoxyadenosine in human retinal DNA by liquid chromatography nanoelectrospray-tandem mass spectrometry
Source: Sci Rep. 2016 Mar 16;6:22375. doi: 10.1038/srep22375 (PMC4793187; doi:10.1038/srep22375)
Supplement: Supplementary Information [file srep22375-s1.pdf]

Supporting Information

Simultaneous determination of 8-oxo-2'-deoxyguanosine and 8-oxo-2'-deoxyadenosine in human retinal DNA by liquid chromatography nanoelectrospray-tandem mass spectrometry

Bin Ma,[†] Meng Jing,[†] Peter W. Villalta,[†] Rebecca J. Kapphahn,[§] Sandra R. Montezuma,[§] Deborah A. Ferrington,[§] and Irina Stepanov*[, †, ‡]

[†]Masonic Cancer Center, [§]Department of Ophthalmology and Visual Neurosciences, and [‡]Division of Environmental Health Sciences, University of Minnesota, Mayo Mail Code 806, 420 Delaware Street SE, Minneapolis, Minnesota 55455, United States

Corresponding Author:

*Phone: (612) 624-4998. Fax: (612) 626-5135. E-mail: stepa011@umn.edu.

Table of Contents

Figure S1	Chromatograms obtained upon analysis of possible artifactual 8-oxo-dG production during the sample preparation	3
Figure S2	Chromatogram of a standard mixture of deoxyuridine, dG, thymidine, 8-oxo-dG, M_1dG and 8-oxo-dA during column chromatography purification	4
Figure S3	Typical chromatograms obtained upon analysis of dG in standard solution and mitochondrial DNA from one donor’s retina.	5

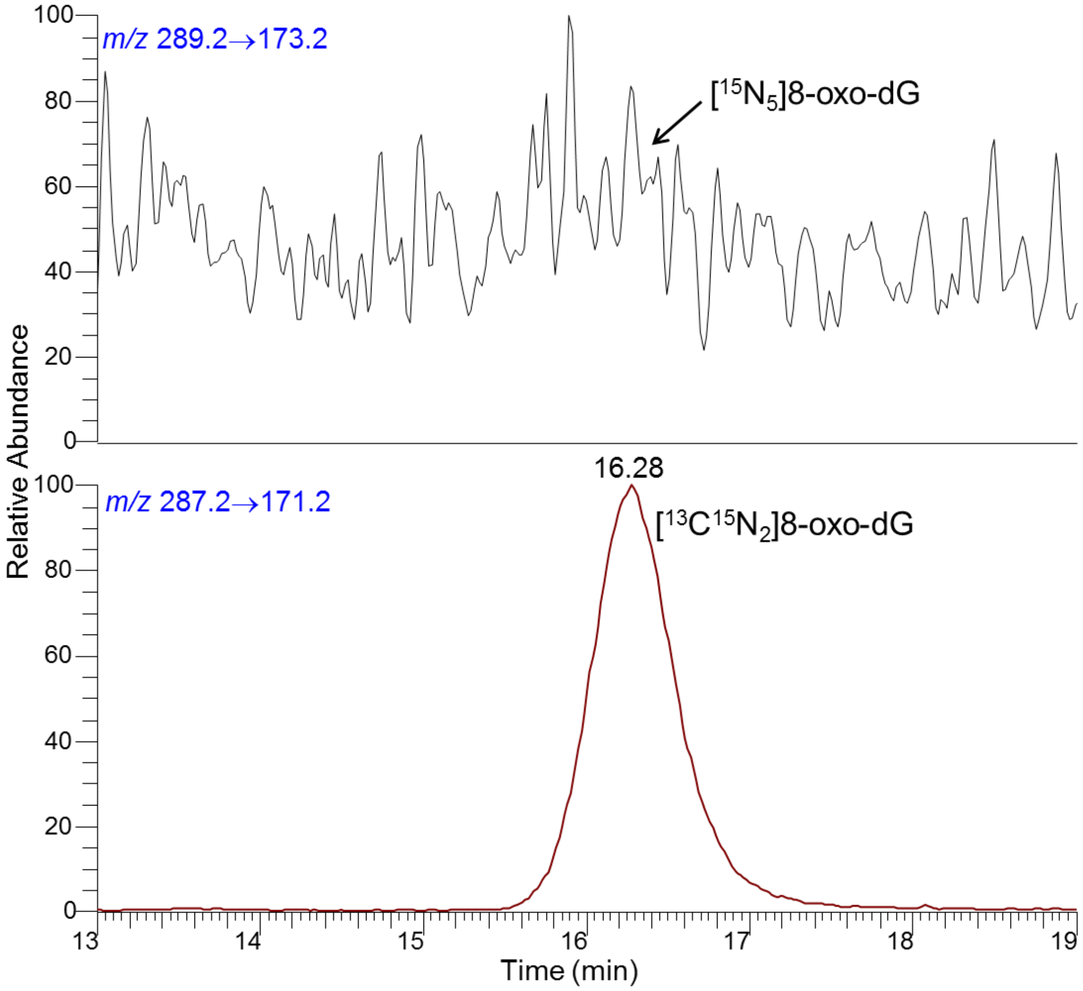

Figure S1. Chromatograms obtained upon analysis of possible artifactual 8-oxo-dG production during the sample preparation. [$^{15}N_5$]8-oxo-dG was not detected.

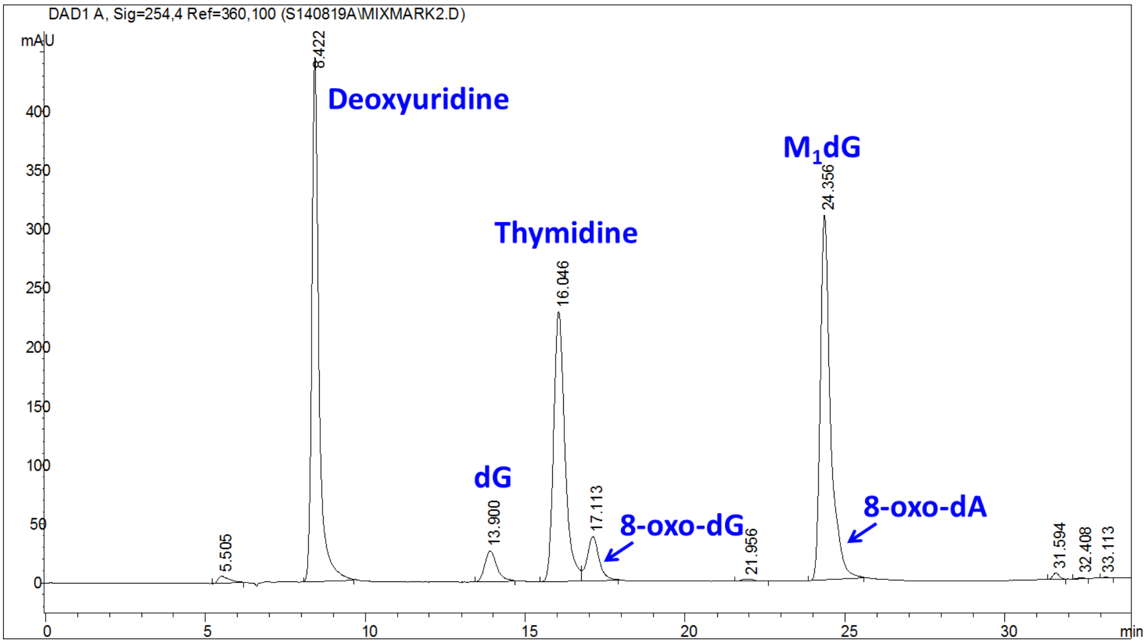

Figure S2. Chromatogram of a standard mixture of deoxyuridine, dG, thymidine, 8-oxo-dG, M_1dG and 8-oxo-dA during column chromatography purification.

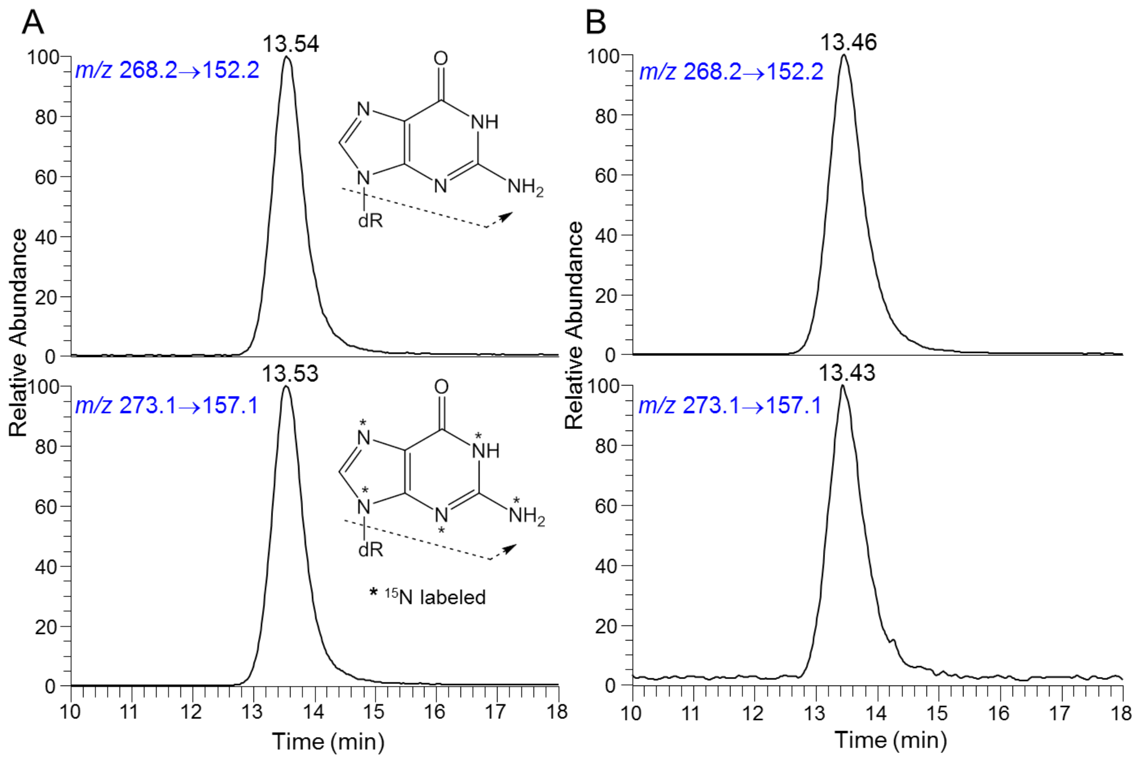

Figure S3. Typical chromatograms obtained upon analysis of dG in (A) standard solution and (B) mitochondrial DNA from one donor's retina.
